# CAPG: comprehensive allopolyploid genotyper

**DOI:** 10.1093/bioinformatics/btac729

**Published:** 2022-11-11

**Authors:** Roshan Kulkarni, Yudi Zhang, Steven B Cannon, Karin S Dorman

**Affiliations:** Department of Agronomy, Iowa State University, Ames, IA 50011, USA; Department of Statistics, Iowa State University, Ames, IA 50011, USA; USDA—Agricultural Research Service, Corn Insects and Crop Genetics Research Unit, Ames, IA 50011, USA; Department of Statistics, Iowa State University, Ames, IA 50011, USA; Department of Genetics, Development and Cell Biology, Iowa State University, Ames, IA 50011, USA

## Abstract

**Motivation:**

Genotyping by sequencing is a powerful tool for investigating genetic variation in plants, but many economically important plants are allopolyploids, where homoeologous similarity obscures the subgenomic origin of reads and confounds allelic and homoeologous SNPs. Recent polyploid genotyping methods use allelic frequencies, rate of heterozygosity, parental cross or other information to resolve read assignment, but good subgenomic references offer the most direct information. The typical strategy aligns reads to the joint reference, performs diploid genotyping within each subgenome, and filters the results, but persistent read misassignment results in an excess of false heterozygous calls.

**Results:**

We introduce the Comprehensive Allopolyploid Genotyper (CAPG), which formulates an explicit likelihood to weight read alignments against both subgenomic references and genotype individual allopolyploids from whole-genome resequencing data. We demonstrate CAPG in allotetraploids, where it performs better than Genome Analysis Toolkit’s HaplotypeCaller applied to reads aligned to the combined subgenomic references.

**Availability and implementation:**

Code and tutorials are available at https://github.com/Kkulkarni1/CAPG.git.

**Supplementary information:**

Supplementary data are available at *Bioinformatics* online.

## 1 Introduction

**Fig. 1.**
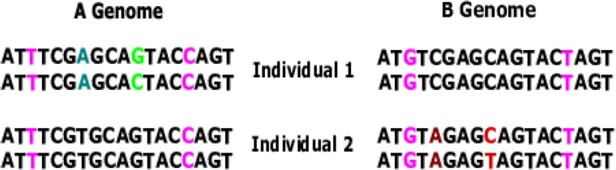
Distinguishing homoeologous and allelic SNPs. Allotetraploid genomes for two individuals, subgenome A (left), subgenome B (right). Pink sites are homoeologous SNPs, different between and constant within subgenomes. Other colored sites are allelic SNPs, green in subgenome A, red in subgenome B. The dark green and brown sites are homozygous. The light green and red sites are heterozygous in one of the individuals (A color version of this figure appears in the online version of this article)

Polyploidy is an important phenomenon, especially in plants, that drives the pace and opportunity for evolution in affected lineages (Soltis and Soltis, 2012; Wendel, 2015). The majority of polyploid plants are allopolyploids (rather than autopolyploids), arising due to interspecific hybridization (Parisod *et al.*, 2010). Allopolyploids include economically important crops such as peanut, wheat, cotton, quinoa and rapeseed. While allopolyploidy is common and consequential, available genotyping methods frequently perform poorly in allopolyploid species (Limborg *et al.*, 2016; Mason, 2015), causing problems for both applied work [e.g. breeding (Clevenger and Ozias-Akins, 2015)] and basic biology (Kulkarni *et al.*, 2020). Figure 1 shows the two classes of Single Nucleotide Polymorphisms (SNPs) in allopolyploids. A homoeologous SNP is an allelic difference between subgenomes that is not segregating within either subgenome. A homologous or allelic SNP is segregating within at least one subgenome. In allopolyploids, SNPs are often misclassified, typically manifesting as an excess of heterozygous calls (Kulkarni *et al.*, 2020; Shirasawa *et al.*, 2016).

Allopolyploids can be genotyped given next-generation sequencing (NGS) reads aligned to a single reference representing both subgenomes along with allele frequencies, rate of heterozygosity, parental genotype or other input. The additional input provides information for assigning reads to their subgenomic source and genotyping. For example, software EGB (Blischak *et al.*, 2018) requires allele frequency estimates from at least one parent species while updog (Gerard *et al.*, 2018) and polyRAD (Clark *et al.*, 2019) assume polyploid individuals are sampled from populations with known genetic structure, such as Hardy–Weinberg equilibrium (HWE). Without such information, the total alternate allele dosage across both subgenomes can be estimated (Blischak *et al.*, 2018) using autopolyploid genotypers, like samtools (Li *et al.*, 2009) or Genome Analysis Toolkit (GATK) (McKenna *et al.*, 2010). Increasingly, however, available subgenome references (Bertioli *et al.*, 2016; Lu *et al.*, 2019; Wang *et al.*, 2019) or genomes of closely related diploid ancestral species (Bertioli *et al.*, 2016; Du *et al.*, 2018) provide direct information for assigning reads. SWEEP (Clevenger *et al.*, 2015) and HAPLOSWEEP (Clevenger *et al.*, 2018), the latter recommended over SWEEP (Peng *et al.*, 2020), use these references to identify allelic SNPs via homozygous individuals at variable sites bracketed by likely homoeologous sites. However, the most common subgenome reference aware method (Peng *et al.*, 2017; Zhou *et al.*, 2014, and M2 in Peng *et al.*, 2020) aligns reads to both subgenomes, keeps uniquely aligned reads and applies a diploid genotyper to each subgenome.

Methods using subgenomic references either use an aligner to imperfectly partition reads to subgenomes or incorrectly process reads from all allopolyploid chromosomes with a diploid genotyper. Instead, we describe the Comprehensive Allopolyploid Genotyper (CAPG), which uses a likelihood to weight read alignments to *both* subgenomes while genotyping individuals from NGS data. Calls are reported in variant call format, with familiar measures, such as genotype likelihood, to gauge statistical support. For samples of individuals, sites are classified as homoeologous SNPs, allelic SNPs within subgenome or invariant. We test the method on simulated data and whole-genome sequencing (WGS) data from two allotetraploids: Peanut (*Arachis hypogaea*) and Cotton (*Gossypium hirsutum*), comparing to GATK on reads partitioned by joint alignment to subgenomic references. HAPLOSWEEP could not run because no haplotypes passed the inclusion criteria. While currently implemented for allotetraploids, CAPG can be extended to higher ploidy levels.

## 2 Approach

### 2.1 Model

Consider a homoeologous genomic region in an allotetraploid individual with A and B subgenomes. We assume reference sequences, with known alignment in the homoeologous region, are available for both subgenomes. Our goal is to genotype the individual in the homoeologous region given whole genome sequencing reads. We align each read, once each to subgenome A and B, producing *n* reads $r_{1},\;r_{2}$, …, $r_{n}$ and quality scores $q_{1},\;q_{2}$, …, $q_{n}$ with *homoeologous alignments* to a site in the homoeologous region. Two alignments are homoeologous at a site if the *same* read base aligns to the homoeologous site in both subgenomes. All other read alignments spanning this site are discarded as likely sequencing or library preparation errors, such as recombination, or reads of paralogous regions, any such reason rendering contradictory alignments. Discarded reads may also reflect genuine alignment ambiguity, particularly around indels, where alignment refinement is warranted (McKenna *et al.*, 2010). CAPG does not genotype indels.

The genotype we wish to call, for example, CC/CT, represents the unordered nucleotides at a site from the maternal and paternal copies of the A subgenome, followed by the unordered nucleotides of the B subgenome. Assuming no more than two distinct nucleotides at the site (for want of better terminology, the major and minor alleles), we can represent the genotype as $M=(m_{1},m_{2})$, where $m_{1},m_{2}\in \{0,1,2\}$ are the numbers of minor alleles in the A and B subgenomes. Given *n* independent reads with homoeologous alignments to a site, we seek the genotype ***M*** with the highest posterior probability,

(1) $$\begin{array}{c} \mathrm{Pr}[M=(m_{1},m_{2})\;|\;r_{1},r_{2},\ldots ,r_{n}]\\ \propto \prod_{i=1}^{n}\mathrm{Pr}\left[R_{i}=r_{i}\;|\;M=(m_{1},m_{2})\right]. \end{array}$$

Conditioning on the homoeologous alignments of the *i*th read and assuming only the true source subgenome $S_{i}\in \{1,2\}$, i.e. which is the true alignment, is unknown, the read likelihood is

(2) $$\begin{array}{c} \mathrm{Pr}\left[R_{i}=r_{i}\;|\;M=(m_{1},m_{2})\right]\\ =\sum_{s=1}^{2}\mathrm{Pr}\left(S_{i}=s\;|\;M\right)[\prod_{\overset{j=1}{j\neq j_{i}}}^{l_{i}}\mathrm{Pr}\left(R_{ij}=r_{ij}\;|\;S_{i}=s\right)]\\ \times \;\mathrm{Pr}\left(R_{ij_{i}}=r_{ij_{i}}\;|\;S_{i}=s,M_{s}=m_{s}\right), \end{array}$$

where *j_i_* is the position in read $r_{i}$ aligned to the site in both alignments and *l_i_* is the aligned length of the *i*th read after removing all insertions, deletions and sites without homoeologous alignments. While read indels are highly informative of subgenomic source because sequencing indels are rare, we neglect them out of concern that unrecognized homoeologous indel variation not present in the references or segregating homologous indel variation would drive the read likelihood. Thus, we solely rely on homoeologous (mis)matches to provide the signal for the subgenomic assignment of reads. When homoeologous SNPs are sparse, the genotype will be called with appropriate uncertainty.

We finish by formulating each probability in the equation. Assuming uniform subgenomic coverage, $\mathrm{Pr}\left(S_{i}=s\;|\;M\right)=0.5$, let *t_r_* be the major and *t_a_* the minor allele at the sites. If *T_i_* is the true nucleotide in the chromosome sequenced in the *i*th read, then under the additional assumption of equal chromosomal coverage, $\mathrm{Pr}\left(T_{i}=t_{r}\;|\;S_{i}=s\right)=1-m_{s}/2$. Therefore,

$$\begin{array}{c} \mathrm{Pr}\left(R_{ij_{i}}=r_{ij_{i}}|S_{i}=s,M_{s}=m_{s}\right)\\ =\sum_{t\in \{t_{r},t_{a}\}}\mathrm{Pr}\left(R_{ij_{i}}=r_{ij_{i}}\;|\;T_{i}=t\right)\mathrm{Pr}\left(T_{i}=t\;|\;M_{s}=m_{s}\right)\\ ={\left(1-e_{ij_{i}}\right)}^{1_{\left\{r_{ij_{i}}=\;t_{r}\right\}}}{\left(\frac{e_{ij_{i}}}{3}\right)}^{1_{\left\{r_{ij_{i}}\neq \;t_{r}\right\}}}\frac{2-m_{s}}{2}\\ +\;{\left(1-e_{ij_{i}}\right)}^{1_{\left\{r_{ij_{i}}=\;t_{a}\right\}}}{\left(\frac{e_{ij_{i}}}{3}\right)}^{1_{\left\{r_{ij_{i}}\neq \;t_{a}\right\}}}\frac{m_{s}}{2}, \end{array}$$

where $1_{\left\{\cdot \right\}}$ is an indicator function, $e_{ij_{i}}=10^{-q_{ij_{i}}/10}$ is the probability of a sequencing error assuming PHRED quality scores (Ewing and Green, 1998) and assuming equal probability of the three possible substitution errors. For read position $j\neq j_{i}$ in read *i* aligned to site *l*, we assume the genotype is homozygous $g_{sl}g_{sl}$, where *g_sl_* is the allele in the *s*th reference genome at site *l*. Thus, $\mathrm{Pr}(R_{ij}=r_{ij}\;|\;S_{i}=s)$ is

(3) $$\begin{array}{c} {\left(1-e_{ij}\right)}^{1_{\left\{r_{ij}\;=\;g_{sl}\right\}}}{\left(\frac{e_{ij}}{3}\right)}^{1_{\left\{r_{ij}\;\neq \;g_{sl}\right\}}}. \end{array}$$

The assumption of homozygosity at other sites may be violated by nearby allelic SNPs or errors in the references, and PHRED errors are not warranted in most datasets. Reference correction and quality score recalibration can help (McKenna *et al.*, 2010), but we leave such efforts to future work.

### 2.2 Genotyping and SNP calling

Genotyping an individual requires identifying the most plausible alleles at each genomic site. SNP calling considers data from *v* multiple individuals to identify variable sites in the population. Modern SNP callers typically assume and estimate a probability distribution, e.g. HWE, for genotypes at a site in a population. Estimating population parameters from the data of multiple individuals improves genotype calling, especially in low-coverage situations (Nielsen *et al.*, 2011). Such an approach is possible in our framework, but for our data, we could not assume HWE, let alone a common source population. Instead, we independently genotype individuals and combine the results to perform SNP calling assuming a uniform (uninformative) prior on the genotypes. We leave it to future work to model and estimate parameters of the population.

#### 2.2.1 Genotyping

Sampled individuals are independently genotyped assuming biallelic sites. For each site in a homoeologous region, we identify the two most common alleles observed among the *n_k_* reads $R_{k}=\{r_{k1},r_{k2},\ldots ,r_{kn_{k}}\}$ with homoeologous alignments to the site in individual *k*, calling the most common allele the *major allele t_kr_* and the second most common allele the *minor allele t_ka_*, breaking ties by the alphabetic ordering of the nucleotides. If there is no second allele observed in the reads with homoeologous alignments, we choose the first alphabetically ordered nucleotide not already denoted the major allele as the minor allele. We then compute the posterior probability of all nine possible allotetraploid genotypes via Eq. (1) and call the genotype as the most likely. We assess the support for heterozygosity at the site in individual *k* and subgenome *g* as the log-likelihood ratio

(4) $$\ln\left[\frac{\mathrm{Pr}(M_{kg}=1\;|\;R_{k})}{\max_{m\in \{0,2\}}\mathrm{Pr}(M_{kg}=m\;|\;R_{k})}\right],$$

where *M_kg_* is the genotype for individual *k* at the site in subgenome *g*.

#### 2.2.2 SNP calling

We also limit SNP identification to biallelic SNPs, involving nucleotides *N*_1_ and *N*_2_. We compute metrics to call homoeologous and allelic SNPs under a uniform prior over all possible genotypes. Since we identify major *t_kr_* and minor *t_ka_* alleles separately for each individual *k*, if the posterior probability of the required genotype is not among the nine computed, we substitute the minimum of the nine. This approximation can be remedied by defining the major and minor alleles from the joint data.

The support for allelic SNPs in subgenome *g* is assessed as

(5) $$\begin{array}{c} -\max_{N\in \{N_{1},N_{2}\}}\sum_{k=1}^{v}[1_{\left\{t_{kr}=N\right\}}\ln\mathrm{Pr}\left(M_{kg}=0\;|\;R_{k}\right)\\ +\;1_{\left\{t_{ka}=N\right\}}\ln\mathrm{Pr}\left(M_{kg}=2\;|\;R_{k}\right)\\ -\;\ln\mathrm{Pr}(M_{kg}={\hat{M}}_{kg}\;|\;R_{k})], \end{array}$$

where ${\hat{M}}_{k}=\left({\hat{M}}_{k1},{\hat{M}}_{k2}\right)$ is the called genotype for individual *k* at the site. The sum allows either major or minor allele to be N, which varies with read coverage. Support for homoeologous SNPs is assessed as

(6) $$\begin{array}{c} \max_{N\in \{N_{1},N_{2}\}}\sum_{k=1}^{v}[1_{\left\{t_{kr}=N\right\}}\ln\mathrm{Pr}\left(M_{k}=(0,2)\;|\;R_{k}\right)\\ +\;1_{\left\{t_{ka}=N\right\}}\ln\mathrm{Pr}\left(M_{k}=(2,0)\;|\;R_{k}\right)\\ -\;\ln\mathrm{Pr}(M_{k}={\hat{M}}_{k}\;|\;R_{k})]. \end{array}$$

## 3 Materials and methods

### 3.1 Implementation

**Fig. 2.**
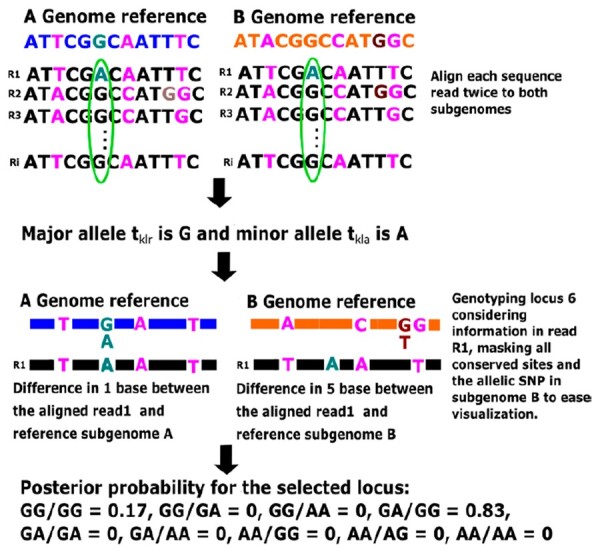
Genotyping an allelic SNP with CAPG. Blue: subgenome A reference sequence; Orange: subgenome B reference sequence; Pink: homoeologous SNPs at sites 3, 8 and 12; Green: segregating allele in subgenome A at site 6; Brown: segregating allele in subgenome B at site 11; Green ellipse identifies the site to genotype in this example, with genotype GA/GG yielding the highest posterior probability of 0.83 (A color version of this figure appears in the online version of this article)

Figure 2 describes the main ideas behind CAPG genotyping. Reads are aligned separately to both subgenomic reference genomes, major and minor alleles for a site are identified, and the likelihood of each read aligned to each subgenome for nine possible genotypes is used to compute the posterior probabilities by Eq. (1). There is opportunity to modify the workflow, from choice of short read aligner to read filtering or skip genotyping sites with, for example, low coverage (details in Supplementary Section S1.1). Worked examples are available at https://github.com/Kkulkarni1/CAPG. In this work, we genotype all sites except indels in the subgenome reference alignment and those with no homoeologous coverage of one or more subgenomes by Supplementary Eq. (S1).

CAPG includes optional *post hoc* filters for allotetraploid genotyping in the face of data artifacts. The model (Section 2.1) assumes equal homoeologous and homologous chromosome coverage, so a false heterozygous call may arise at a site with an unusually high sequencing error rate, an amplified PCR error or biased sequencing of one subgenome. Though the resulting high frequency of the alternate allele is inconsistent with variation due to sequencing errors, it is unlikely to exactly match 50% of the subgenomic coverage. While the assumption of equal chromosomal coverage is not always valid (Gerard *et al.*, 2018; Rozowsky *et al.*, 2011), we derive and implement a likelihood ratio test (details in Supplementary Section S1.1.2) of equal homologous coverage in the presence of unequal homoeologous coverage, which can be used to screen heterozygous calls for some error and coverage artifacts.

### 3.2 Simulation

We compared CAPG and GATK (McKenna *et al.*, 2010) on their ability to provide valid input, e.g. normalized PHRED-scaled likelihood (PL), for correctly identifying heterozygous sites, allelic SNPs and homoeologous SNPs on simulated WGS data. Simulation details are in Supplementary Section S1.3. Briefly, we simulated 100 000 sites in 50 individuals, with about 1 allelic SNP per 100 sites and homoeologous SNP rate $r_{h}\in \{0.005,0.007,0.01\}$. We genotyped each site in each individual based on simulated paired-end reads of length 150 from fragments of mean length 300 ± 10 and subgenomic coverage rate c∈{10,20,40}.

#### 3.2.1 Running CAPG

Simulated reads were aligned separately against the simulated reference subgenomes using BWA-MEM2 (Vasimuddin *et al.*, 2019) with default settings. Some sites (<0.1%) were dropped from heterozygosity calling because of no read coverage; two sites were dropped from SNP calling because of no read coverage across all 50 individuals at coverage level *c *=* *10 (details in Supplementary Table S1). These sites are excluded from all presented results. PR curves were plotted based on the CAPG metrics of Eqs. (4)–(6) for evaluating heterozygosity, allelic SNPs and homoeologous SNPs. The metric (6) is sometimes −∞, which is replaced in plots with $2r_{\left(2\right)}-r_{\left(3\right)}$, where $r_{\left(i\right)}$ is the *i*th smallest metric value. Sites are sometimes subsampled as indicated in figure legends to ease viewing.

#### 3.2.2 Running GATK

We aligned reads to the joint tetraploid reference A and B subgenomes, using BWA-MEM2, followed by discard of secondary alignments. GATK’s HaplotypeCaller was used to separately genotype each subgenome using default parameters. Quantities equivalent to CAPG metrics Eqs. (4)–(6) [see Supplementary Eqs. (S3)–(S5)] were computed for all homoeologous positions in the subgenomic reference alignment.

### 3.3 Validation on real data

We downloaded WGS resequencing data from 14 peanut and 9 cotton germplasms of diverse origin (Clevenger *et al.*, 2017; Fang *et al.*, 2017; Pan *et al.*, 2020) (accessions listed in Supplementary Tables S3 and S4). We identified genic regions in the genome annotation files of the peanut Tiffrunner assembly (Bertioli *et al.*, 2019) and the cotton TM1 assembly (Li *et al.*, 2015), downloaded from PeanutBase (Dash *et al.*, 2016) and NCBI, respectively. We selected 1,000 genic homoeologies found using BLAST (Altschul *et al.*, 1990) with 95–98% sequence similarity and alignment length over 1,000 bps. These sequences were genotyped using CAPG as for simulation, but *post hoc* filtered for calls with expected coverage of either subgenome below eight (Minimum coverage in Supplementary Section S1.1.1), no subgenome mismatches within read distance (Identifiable in Supplementary Section S1.1.2), or for heterozygous calls, rejection of the equal homologous coverage hypothesis at significance level 0.05 (equal homologous coverage test in Supplementary Section S1.1.2). GATK was run as for simulation data, except indel variants were removed by bcftools, calls with fewer than eight reads per either subgenome or no subgenomic mismatches within read distance, and heterozygous calls rejected at significance level 0.05 via a likelihood ratio test of equal homologous coverage using the allele depth data (SAM tag AD) reported by HaplotypeCaller were discarded.

## 4 Experimental results

### 4.1 Simulation

To verify CAPG performance, we simulated allotetraploid data while varying subgenomic read coverage (*c*) and homoeologous rate (*r_h_*). As expected, performance of CAPG improves with higher coverage and more homoeologous SNPs. The details are provided in the Supplementary Section S2.1.

**Fig. 3.**
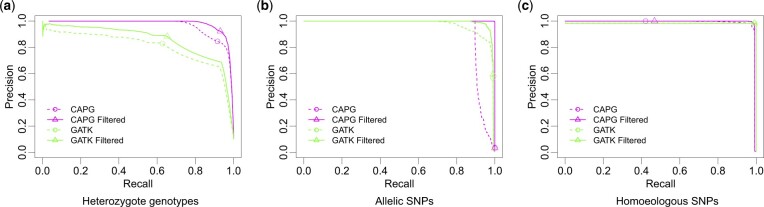
PR curves for CAPG and GATK on simulated data. Performance of CAPG and GATK metrics to identify (**a**) heterozygous sites in subgenome A, (**b**) allelic SNPs in subgenome A and (**c**) homoeologous SNPs when coverage *c *=* *10 and homoeologous rate $r_{h}=0.007$. Heterozygous data are subsampled with all true positive sites and 100 000 randomly sampled true negative sites. Circles/triangles represent the threshold value (0), a liberal choice (high recall, low precision, and also see Supplementary Fig. S4) for genotyping heterozygotes and progressively more conservative with sample size for SNP calling

We also compared the performance of CAPG with benchmark GATK (McKenna *et al.*, 2010), using reads assigned to subgenome by alignment, on ability to detect heterozygosity and predict SNPs. Performance as a function of coverage and homoeologous rate is detailed in Supplementary Section S2.1. Here, we report results for the simulation with $r_{h}=0.7\%$ and *c *=* *10. Figure 3a shows that CAPG better detects heterozygosity than GATK. For allelic SNPs (Fig. 3b), CAPG is only superior to GATK at high precision. At the threshold where the PR curves cross, there are 52 allelic SNPs in subgenome A not called by CAPG and all are nonidentifiable, having no homoeologous SNP within read length distance. The CAPG metric is appropriately low and provides equal support for an allelic SNP in subgenome B. The GATK metric also reflects ambiguity, with about half (30 versus 22) showing stronger support for an allelic SNP in subgenome B, where the site is invariant. The difference is that the GATK metrics are larger; 30 of the 52 unidentifiable allelic SNPs are already called at the threshold where the PR curves cross. This strong signal is an artifact of the complete confidence placed in the read assignments, which also results in high rates of false heterozygous calls (Fig. 3a). After removing all nonidentifiable sites, both PR curves improve (solid lines in Fig. 3), but CAPG is superior, only misclassifying two identifiable allelic SNPs using a threshold of 21.1. Finally, CAPG homoeologous SNP calling at the default threshold reaches equal precision but lower recall than GATK (Fig. 3c). The increased uncertainty of CAPG is because it does not use the genotyped site to assign reads, a fact we discuss further in the next section.

**Fig. 4.**
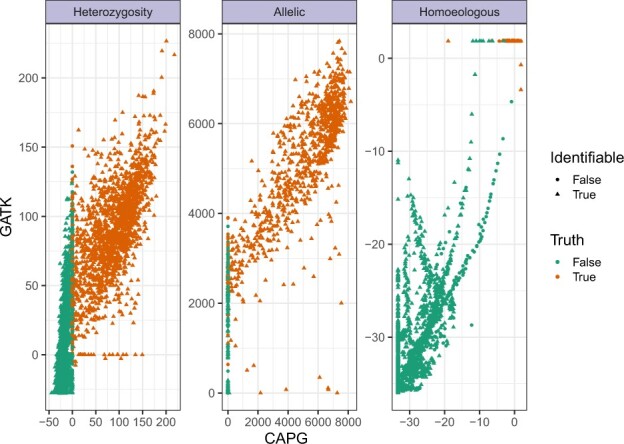
Comparing CAPG and GATK metrics in simulation. Scatter plot of heterozygosity, allelic SNP and homoeologous SNP metrics on simulated data with coverage *c *=* *40 and homoeologous rate $r_{h}=0.007$. Homoeologous metrics (Eq. (6) and Supplementary Eq. (S5)) are transformed via Box–Cox transformation $-\frac{{\left(-x+0.1\right)}^{\lambda }-1}{\lambda },\;\lambda =0.2$ to avoid overplotting at upper right, and the stack of points on the left represents a transformation of CAPG metric value −∞ (see Section 3). There remain overplotted true homoeologous SNPs, but all true negatives (green) are visible after transformation. In addition to subsampling done for heterozygosity (see Fig. 3), we further subsample to avoid excess overplotting, keeping all points with CAPG metric > 0 for heterozygosity or finite for homoeologous SNPs and subsampling 10% of all other points

A scatter plot of the CAPG and GATK metrics underlying the PR curves, but for coverage *c* = 40, is shown in Figure 4, with true status indicated in color. Non-identifiable sites are plotted as circles; these sites form the lower tier of points in the homoeologous facet. CAPG metrics are much closer to linearly separable than GATK metrics. GATK sometimes indicates strong support for false positive SNP calls, and while CAPG is not perfect, when it makes a false call, the metric indicates borderline support.

### 4.2 Real data

We collected 14 peanut and 9 cotton accessions. After whole genome alignment, average read coverage per site per subgenome was 25 (range 15–40 across accessions) for peanut and 12 (range 9–21) for cotton. We genotyped 1,000 selected gene sequences with both CAPG and GATK. Selection of thresholds for calling heterozygous genotypes, allelic SNPs and homoeologous SNPs is described in Supplementary Section S2.2.

SNPs were distributed throughout the selected genic sequences in both subgenomes of peanut and cotton (data not shown). Table 1 shows 1.7% of genotyped sites in peanut are likely homoeologous SNPs, far fewer are likely allelic SNPs (0.016%, distributed equally in both subgenomes), and even fewer sites are convincingly heterozygous, suggesting the sample consists of largely inbred individuals. We found a higher homoeologous (2.5%) and allelic (0.08%) SNP rate in cotton (Table 2), consistent with previous findings (Bertioli *et al.*, 2016; Fang *et al.*, 2017). The chosen threshold probably excludes some true allelic SNPs. While metrics for allelic SNPs and monomorphic sites are well-separated in simulation (Supplementary Fig. S4h and k), the two groups clearly overlap in real data (Supplementary Fig. S4b and e). Nevertheless, there are metrics in both species indicative of allelic SNPs. Higher coverage, larger samples or biological verification may confirm the predicted allelic SNPs from this study.

**Table 1.** SNPs identified by CAPG from 1,000 selected gene sequences among 14 peanut accessions

Chrom. A/B	No. of genes	Total bps	Typed bps	SNP type			Het.	
				A	B	H	A	B
Chr1/11	124	382 667	374 483	27	41	6451	10	28
Chr2/12	59	191 031	187 186	10	10	3474	0	1
Chr3/13	163	504 002	495 213	38	36	8381	13	11
Chr4/14	77	248 104	240 961	48	55	4030	30	20
Chr5/15	129	388 839	380 297	18	28	6276	0	8
Chr6/16	103	335 647	329 849	19	26	5434	0	13
Chr7/17	60	195 670	190 549	26	10	3578	1	1
Chr8/18	94	284 517	273 462	21	10	4580	3	0
Chr9/19	113	380 543	369 665	32	17	6378	9	1
Chr10/20	78	265 632	259 947	16	13	4458	5	2
Total	1000	3 176 652	3 101 612	255	246	53 040	71	85

Chrom., subgenome A/subgenome B; Total bps, total sites in selected genes; Typed bps, genotyped sites; A, subgenome A; B, subgenome B; H, homoeologous SNP calls; Het., heterozygous sites.

**Table 2.** SNPs identified by CAPG from 1,000 selected gene sequences among 9 cotton accessions

Chrom. A/D	No. of genes	Total bps	Typed bps	SNP type			Het.	
				A	D	H	A	D
Chr1/14	74	188 535	170 513	79	94	4517	9	1
Chr2/15	47	129 470	101 284	42	34	2966	1	3
Chr3/16	57	144 060	127 882	30	58	3188	2	2
Chr4/17	34	88 886	70 335	38	45	2142	20	36
Chr5/18	164	470 043	403 721	167	197	10 591	11	9
Chr6/19	62	160 249	150 071	40	39	3680	1	2
Chr7/20	73	186 525	173 147	56	125	4283	8	2
Chr8/21	80	198 353	184 720	73	61	4624	8	3
Chr9/22	72	197 435	191 502	80	66	4497	8	28
Chr10/23	74	186 009	152 265	79	59	4396	9	2
Chr11/24	108	314 290	289 887	69	67	6880	1	2
Chr12/25	95	231 822	220 009	72	96	5255	6	0
Chr13/26	60	162 958	143 942	94	46	3640	14	2
Total	1000	2 658 635	2 379 293	919	987	60 659	98	92

Chrom., subgenome A/subgenome D; Total bps, total sites in selected genes; Typed bps, genotyped sites; A, subgenome A; D, subgenome D; H, homoeologous SNP calls; Het., heterozygous sites.

**Fig. 5.**
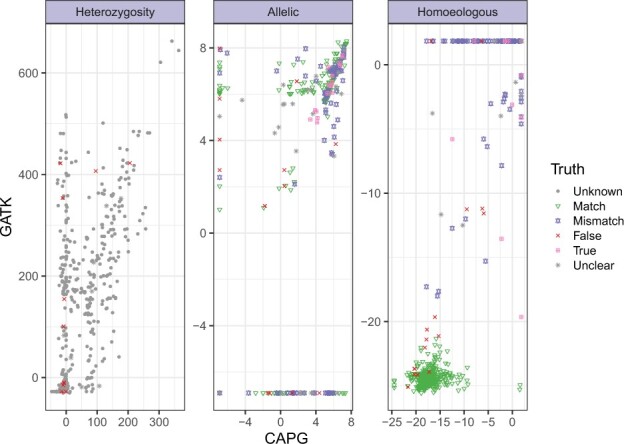
Comparing CAPG and GATK metrics in real peanut data. Scatter plot of metrics for heterozygosity, allelic and homoeologous SNPs. We examine alignments to confirm (True: pink boxed ‘x’), reject (False: red plus) or fail to resolve (Unclear: gray star) a small selection of sites. Otherwise, the heterozygosity status is unknown (gray circle), but we indicate if there is a subgenomic reference nucleotide match (green triangle) or mismatch (purple square) in the allelic and homoeologous facets. After including all hand-verified sites, a stratified sample was taken to over-sample likely heterozygous calls by either method, so 50% of sampled sites have CAPG or GATK metrics above the 99.5th percentile. For allelic SNP metrics, we sampled 25% sites with subgenomic mismatch, 25% sites with either CAPG or GATK metric above the 99.5th percentile, 25% sites with subgenomic match and 25% with both metrics below the 99.5th percentile. For homeologous SNP metrics, we sampled 50% sites with subgenomic mismatch and 50% with subgenomic match and low metrics by CAPG and GATK. For an unbiased view of the metrics, see Supplementary Figures S7–S9

Scatterplots of unfiltered CAPG and GATK metrics for the peanut data show greater variability than simulation data (Fig. 5). There is positive association between CAPG and GATK in all three metrics, but the association is weak (Spearman correlation ρ<0.4) for the SNP metrics (Supplementary Table S5). We sampled several identifiable sites and manually genotyped the read alignments (plot symbols). As for simulated data, there is a pattern of sites with CAPG heterozygosity metric near 0 and increasing GATK metric up the *y*-axis, likely false heterozygous calls by GATK and confirmed in visual spot checks (red crosses). However, we also could not confirm heterozygosity for sites where both methods agree (upper right), mostly because paralogous reads mapping to the locus obscured the signal (gray stars). We could manually identify and ignore paralogous reads at two sites to find no heterozygosity (red crosses), but neither method can automatically remove paralogous reads. CAPG and GATK strongly disagree on a proportion of sites in the upper left (0.4%) and lower right (3%) of the allelic SNP plot. Causes for sites in the lower right include aligned paralogous reads, mismatches between the subgenomes not recapitulated in the reads, or nearly non-identifiable sites. The latter two kinds of sites also often appear in the upper swath of the homoeologous metric plot, where GATK is certain but CAPG is uncertain in the homoeologous SNP call. These sites tend to be subgenomic mismatches (purple points), information used to separate the reads passed to GATK but ignored by CAPG for read assignment. In our view, CAPG metrics reflect appropriate ambiguity in these cases. In the upper left, GATK is misled by mismapped reads, low levels of an alternate allele with uncertain provenance, and supplementary alignments. Our filters remove many (62% upper left, 92% lower right) of these cases (Supplementary Fig. S6).

We find evidence that CAPG metrics are more tightly associated with other evidence of true SNPs. CAPG metrics better correlate with manually assessed truths (Supplementary Table S5), but while the sampling strategy for sites to assess was neutral, it was not balanced, so we also examined associations in unsampled data. Mismatches between the reference subgenomes should occur at all homoeologous SNPs and some allelic SNPs, so a mismatch can serve as a noisy label for either SNP. PR curves (Supplementary Fig. S10) and numeric associations (Supplementary Table S5) demonstrate the CAPG metric better discriminates mismatch-identified allelic SNPs but not homoeologous SNPs. GATK’s apparent superior performance on homoeologous SNPs is an artifact of using the site itself to partition reads. Examination of allelic and homoeologous SNP metrics in Supplementary Figures S8 and S9 suggests some subgenomic mismatches are actually allelic SNPs. Finally, we expect more extreme (definitive) metrics at identifiable sites. We used Levene’s statistic to measure spread in the metric, confirming the heterozygous metric (CAPG $5\times 10^{4}$ versus GATK $1\times 10^{2}$), the allelic metric (CAPG 0.05 versus GATK 0.01), but not the homoeologous metric (CAPG 73 versus GATK 85) are more extreme at identifiable sites in CAPG. A full discussion of these results and more is in Supplementary Section S2.2.1.

## 5 Discussion

We propose a likelihood-based genotyper, CAPG, to accurately call genotypes and SNPs in allotetraploids. We have shown that CAPG is better at identifying SNPs in both simulation and real data than the benchmark GATK applied to reads split by alignment to reference subgenomes. We now discuss the advantages and limitations of CAPG.

### 5.1 Likelihood-based genotyping

Li (2011) has shown the value of likelihood methods for genotyping and calling SNPs, and it is logical to extend such models to genotype allopolyploids and account for the unknown subgenomic source of the read. To apply the model, we condition on independent alignments of each read against both reference subgenomes. We use the likelihood to assess their relative support, avoiding the approximate choice between homoeologs made by short-read aligners. A complication is that CAPG obtains joint estimates of the allotetraploid genotype, not independent estimates of the two homoeologous diploid genotypes, which necessitates a moderate amount of post-processing to merge into traditional genotyping pipelines. To encourage genotyping at the allotetraploid level, we provide metric scores for allelic and homoeologous SNP calling across the sample. We also provide a formal test of equal coverage of homologous chromosomes that can be used to screen heterozygous calls.

Li (2011) and others have modeled the genotype proportions in the population during variant discovery, and CAPG can be extended to consider such hierarchical models. Our demonstration genotypes individuals independently and then combines the genotyping metrics *post hoc*, essentially placing a uniform prior on plausible genotypes (Nielsen *et al.*, 2011). This approach is reasonable for our real data examples since they include individuals with unknown relationships, where it is unclear what assumptions to impose about the population.

Our presentation and software focus on allotetraploids, but extension to higher ploidies is straightforward. Ambiguous genotypes are increasingly probable at high ploidy. An allelic SNP linked to the alternate allele at homoeologous SNP (0, 2, 2) is either in subgenome B or C, and CAPG would appropriately communicate the uncertainty.

### 5.2 Model limitations

As expected, the performance of our method declines with decreasing coverage. When appropriate, inclusion of a hierarchical population model can increase power, but with advances in NGS technologies, it should also be possible to obtain sufficient coverage.

All methods require homoeologous sites to assign reads to subgenomes, so accuracy declines with short reads, low homoeologous rate or poor subgenomic references. Competitive alignment against the joint reference can recapitulate support using GATK (not CAPG) for homoeologous sites already represented in the subgenomic references. Generally, GATK predisposes homoeologous SNPs at all subgenomic mismatches, even when they are actually allelic SNPs or errors.

In repetitive genomes, reads from paralogous sources lead to excess evidence of heterozygosity. *Post hoc* tests of equal homologous coverage in heterozygotes can eliminate some such false calls. It is also possible to check for evidence of more than four haplotypes at a locus, for example, using denoisers (Peng and Dorman, 2021) to identify and remove paralogous reads or entire contaminated regions. Such an approach was successful when applying CAPG to amplicon sequences (data not shown).

Both methods condition on alignments of reads to the subgenomic references and of subgenomic references to each other. Incorrect subgenomic read assignment disrupts genotyping by GATK, but both methods may detect false signal around indels when there are multiple plausible alignments. It is possible to refine read alignments prior to genotyping, as has shown promise (McKenna *et al.*, 2010). Incorrect subgenomic reference alignments would align non-homoeologous read positions, inducing errors in allelic and homoeologous SNP calling. It is worth investing in good references and alignments. Model modifications could account for known reference deficiencies and computationally intense methods could integrate over uncertainty in the alignment.

### 5.3 Biological significance

Accurate prediction of heterozygosity, allelic SNPs and homoeologous SNPs is important for basic biology—for example, in evolutionary studies—and the applied science of plant breeding and other applications. Since only allelic SNPs segregate, it is clearly important to be able to distinguish allelic and homoeologous SNPs, but accurate genotyping also improves our ability to study gene gain or loss after polyploidization, major structural rearrangements or conservation between homoeologous chromosomes, and functional divergence of polyploids from diploids. From a crop improvement viewpoint, identifying functionally conserved homoeologs can help elucidate the genetic basis for traits of interest.

## Supplementary Material

btac729_Supplementary_Data

## Data Availability

No new data were generated or analysed in support of this research.
